# A Call for a Change in Policy Regarding the Necessity for SDE Tests to Validate the Veracity of the Outcome of Enantioselective Syntheses, the Inherent Chiral State of Natural Products, and Other Cases Involving Enantioenriched Samples

**DOI:** 10.3390/molecules26133994

**Published:** 2021-06-30

**Authors:** Jianlin Han, Roman Dembinski, Vadim A. Soloshonok, Karel D. Klika

**Affiliations:** 1 Jiangsu Co-Innovation Center of Efficient Processing and Utilization of Forest Resources, International Innovation Center for Forest Chemicals and Materials, College of Chemical Engineering, Nanjing Forestry University, Nanjing 210037, China; hanjl@njfu.edu.cn; 2 Department of Chemistry, Oakland University, 146 Library Drive, Rochester, MI 48309, USA; dembinsk@oakland.edu; 3 Department of Bioorganic Chemistry, Centre of Molecular and Macromolecular Studies, Polish Academy of Sciences, Sienkiewicza 112, 90-363 Łódź, Poland; 4 Department of Organic Chemistry I, Faculty of Chemistry, University of the Basque Country UPV/EHU, Paseo Manuel Lardizábal 3, 20018 San Sebastián, Spain; 5 IKERBASQUE, Basque Foundation for Science, Alameda Urquijo 36-5, Plaza Bizkaia, 48011 Bilbao, Spain; 6 Molecular Structure Analysis, German Cancer Research Center (DKFZ), Im Neuenheimer Feld 280, D-69120 Heidelberg, Germany

We wish to draw attention to an important issue concerning scientific practice with regard to enhancing the quality of publications in *Molecules* (as well as for other journals). The concern regards the phenomenon of the self-disproportionation of enantiomers (SDE) [1], a pervasive and omnipresent process [2,3] that occurs whenever a scalemic sample is fractionated during any application of an *achiral* physicochemical process such as recrystallization, sublimation [4], chromatography [5], rotary evaporation [6], or even distillation [7]. As a consequence of the SDE, the enantiomers are distributed disproportionately across fractions, i.e., the enantiomeric excesses (ee’s) of the fractions vary though the ee in total remains unchanged from the original sample. Thus, no matter how accurately ee’s are measured, they will not reflect the outcome of an enantioselective synthesis or the inherent chiral state of a natural product if appropriate care is not taken with regard to physicochemical processes applied prior to ee measurement due to the SDE phenomenon.

An obvious upshot of the SDE is that researchers, as evidenced in many cases, unwittingly and erroneously report the ee of their product from an enantioselective synthesis, or an isolated natural product, etc., if they are not at pains to address the problem of the SDE. Indeed, any study involving physicochemical processes applied to chiral compounds can fall victim to this problem unless known or proven to only involve either racemates or enantiopure samples [2]. Possible means to circumvent deleterious impacts of the SDE include involving only conditions that suppress the magnitude of the SDE to below a level that is considered negligible and the obvious courses of completely avoiding fractionation of the sample during purification or isolation steps or avoiding the issue altogether by measuring the sample ee prior to the application of any physicochemical process concomitant with fractionation. However, as is often the case, fractionation is frequently necessitated and unavoidable, e.g., in chromatography [2,3], and measuring the sample ee prior to sample clean-up may not be conducive to the well-being of the analytical system, e.g., chiral LC and GC columns, or those otherwise compromised by the presence of other chemical components. Thus, as has been recommended in a recent publication [8], one can only be confident that the SDE is not unduly perturbing the sample ee by performing SDE tests to gauge the susceptibility of a compound(s) to the SDE under the applied conditions and affirming that perturbation of the sample ee is indeed negligible to the purpose at hand. The authors of the recommendation for conducting SDE tests, duly accompanied by a statement addressing the issue of possible perturbation of the ee due to the SDE, consider it necessary and critical for SDE tests to be made mandatory and become part of editorial policy for work involving, or potentially involving, the fractionation of scalemic samples.

Though conducting SDE tests slightly increases the demands placed upon the authors of a work, the intended gains of doing so are unquestionable:

- Improvement in the quality of research and enabling more reproducible and reliable results;
- Mitigation of poor, erroneous, or fraudulent science;
- Greater appreciation of the work by readers leading to improved ideas and methods by others; and
- Reduction in confusion regarding the results and/or unnecessary correspondence to clear up trivial aspects that are simply handled by inspection of the data.

The essence of conducting SDE tests is to enhance the trust and veracity of the results for work involving scalemic samples and to elevate the quality and reliability of publications. Though significant changes in editorial policy are relatively uncommon, the scientific necessity for doing so in this case is clear. Indeed, there is plenty of precedent for what we are suggesting with only a small amount of reflection. One could go back in time and see the implementation of elemental analysis (simply combustion analysis originally) required as proof of product purity, and indeed structure. However, as new technologies develop, various demands for proof of purity and structure and simply compound characterization, though not necessarily made redundant, can diminish, or appropriately be relaxed. Prime examples in this respect are HR-MS and HPLC supplementing elemental analysis with similar declines in demand for IR spectra, mp’s, and bp’s, however lamentable they may be [9]. Nevertheless, new techniques as well as supplanting traditional methods can also introduce new additional tests. One can also consider the seismic implementation of supporting materials associated with present-day publications containing relevant data such as copies of spectra, chromatograms, etc. The effects of supporting materials have been profound, leading to greater clarity, transparency, reliability, and reproducibility of research results, making them undoubtedly worthwhile despite the additional, and sometimes considerable, effort involved. Thus, as new technologies develop or new knowledge is acquired, it becomes appropriate to adjust accordingly the necessary demands for proof of success. The SDE is very much a case of new knowledge influencing the change in demands, concomitant with technological advances, viz. greater sensitivity and means to measure ee.

As a consequence, the recommendation for conducting mandatory SDE tests has come as a result of the maturation of the study of the SDE phenomenon. Systematic research into the SDE over the last two decades has revealed [2,3] that the SDE phenomenon is ubiquitous in terms of the types of organic molecules involved, is omnipresent, and can be expected for practically all physicochemical conditions/techniques, thus resulting in manifestation of the SDE with a consequent effect on reported experimental data. Objectively, the rational conclusion that one can draw from the wealth of data on the SDE is that mistakes in reported stereochemical outcomes are simply unavoidable, unless appropriate care is taken in regard to the SDE. Thus, the suggested change in editorial policy requesting authors to acknowledge the problem of the SDE, for example, by referring to appropriate citations [8,10,11] and to conduct appropriate tests, is a natural evolution and progression borne out of increased knowledge and technical capability which continues the growth and development of the craft of stereochemistry.

It is worth pointing out that researchers can be judicious in selecting which and how many tests are appropriate and in conducting, at the very least, the minimal necessary. As has been pointed out [8], normally not much additional work is required as not every compound in a set needs to be tested, perhaps only the one most likely to be conducive to expressing the SDE under the applied process and for conditions which are also most likely to be conducive towards the SDE occurring in a high magnitude consistent with the applied conditions. For example, compounds possessing SDE-phoric groups (functional groups which, when present in a compound, render the compound particularly prone to expressing the SDE [12]) or those which are particularly polar and are thus susceptible to expressing the SDE via normal-phase chromatography [2,5,8], while volatile compounds are susceptible to expressing the SDE [2,8] via sublimation [4], during rotary evaporation [6,13], or upon long-term storage [14,15,16,17]. Thus, the judicious selection of the most polar and/or volatile compounds in a set together with the physicochemical process and conditions, as appropriate, can expedite the evaluation process. However, not only is avoidance of error a possible beneficial outcome and a clear incentive to conduct SDE tests, but SDE observations of high magnitude or unusual occurrence can contribute to the work (as well as the study of the SDE in general) and may well constitute a study in their own right; a highlight example being the SDE examination of the most infamous of drugs, thalidomide [18]. As was stressed in the paper by J. Han, et al. [8], “it is impossible to find any scientifically based argument against the proposal to improve the quality of reported data, credibility of research, and public perception of science as a self-correcting entity”.

## Data Availability

Not applicable.
